# Electron microscopy of desmosomal structures in the pemphigus human skin organ culture model

**DOI:** 10.3389/fmed.2022.997387

**Published:** 2022-11-14

**Authors:** Uta Katharina Radine, Valéria Bumiller-Bini Hoch, Angelica B. Winter Boldt, Detlef Zillikens, Ralf J. Ludwig, Christoph M. Hammers, Matthias Klinger, Jennifer E. Hundt

**Affiliations:** ^1^Lübeck Institute of Experimental Dermatology, University of Lübeck, Lübeck, Germany; ^2^Laboratory of Human Molecular Genetics, Department of Genetics, Federal University of Paraná, Curitiba, Brazil; ^3^Department of Dermatology, University of Lübeck, Lübeck, Germany; ^4^Center for Research on Inflammation of the Skin, University of Lübeck, Lübeck, Germany; ^5^Institute of Anatomy, University of Lübeck, Lübeck, Germany

**Keywords:** pemphigus, human skin organ culture, desmosome, electron microscopy, desmoglein

## Abstract

Pemphigus is a chronic autoimmune skin blistering disease, characterized by acantholysis and by the production of autoantibodies directed against the structural desmosomal proteins desmoglein 1 (DSG1) and/or DSG3. Model systems allow the identification and testing of new therapeutic targets. Here, we evaluated ultrastructural desmosomal morphology in the human skin organ culture (HSOC) model injected with either anti-desmoglein (DSG) 1/3 single-chain variable fragment (scFv, termed Px4-3), Staphylococcus aureus exfoliative toxin (ETA) as a reference and positive control, and normal human IgG as a negative control. Each experimental condition was evaluated in abdominal skin biopsies from five different donors. After 24 h of incubation, we processed the samples for histological and ultrastructural electron microscopy analyses. We found that Px4-3 or ETA induced a loss of desmosomes and increased interdesmosomal widening, similar to patient skin biopsies and other pemphigus models. Thus, we propose the HSOC pemphigus model as an attractive tool to unravel novel therapeutic targets.

## Introduction

Pemphigus are rare, potentially life-threatening, chronic autoimmune skin blistering diseases, with pathogenic autoantibodies mainly directed against the structural desmosomal proteins desmoglein 1 (DSG1) and/or DSG3 (1). Pemphigus foliaceus (PF) presents autoantibodies against DSG1, and mucocutaneous-type pemphigus vulgaris (PV) autoantibodies against DSG3. Mucocutaneous pemphigus presents reactivity against both autoantigens (1, 2). DSGs are cadherin-type Ca2+-dependent transmembrane adhesion molecules (3). Anti-DSG1/3 IgG binding causes acantholysis with desmosomal splitting and keratinocyte separation, hallmarked by intraepidermal split formation, clinically flaccid blisters and secondary erosions (1). Following DSG1/DSG3 redistribution, desmosomal splits occur in the mucosa and skin suprabasal stratum in PV, but solely in the skin stratum granulosum in PF (3, 4). Corticosteroids are the standard pemphigus treatment (5). The anti-CD20 antibody rituximab, combined with corticosteroids, further induces complete remission off-therapy within 24 months in 89% of patients (6). Due to the extended time to achieve remission, the necessity of new therapeutic options remains. Model systems allow the identification and testing of new therapeutic targets. In pemphigus, *in vitro* models (7) and mouse models (8, 9) can be used to that end. While the aforementioned *in vitro* models duplicate certain aspects of pemphigus pathogenesis, mouse models are better suited to assess the impact of an *in vivo* intervention. Mouse models of pemphigus, however, are hampered by the relatively complex experimental procedures (8, 9) or by the differences in DSG expression patterns between mice and men (10). Organ skin models are being increasingly used to overcome these limitations and to implement the replace, reduce, and refine (3R) principles of animal research, including to replace animal experiments by appropriate alternatives (11–14). We recently developed a highly standardized human skin organ culture (HSOC) model of pemphigus using skin from donors of elective surgery (15). Here, a bi-specific anti-DSG1/DSG3 single-chain antibody variable fragment (scFv) binding to both DSG1 and 3, termed Px4-3 (16, 17), is injected into human skin. This consistently induces intraepidermal splits and the model is amendable for therapeutic interventions. Indeed, we recently used this model to identify new therapeutic targets to block acantholysis in pemphigus (18). To obtain additional insights into the mechanisms of how Px4-3 induces split formation, we evaluated the ultrastructural morphology of desmossomal dissociation in the HSOC model. As a reference and positive control, *Staphylococcus aureus* exfoliative toxin (ETA), (Toxin Technology, Sarasota, Fl, USA) mimicking DSG1 autoantibodies-mediated effects (8), was injected into the human skin. Next, we investigated the alterations in interdesmosomal widening, desmosome number and length induced by either Px4-3 or ETA.

## Materials and methods

**FIGURE 1:**
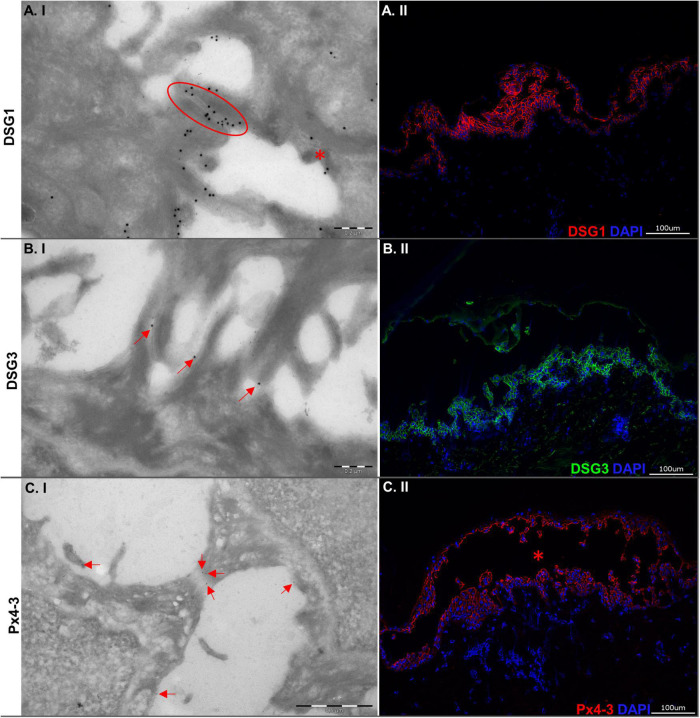
Ultrastructural and immunopathological characterization of the human skin organ culture model for pemphigus vulgaris. **(A.I)** Electron micrograph showing desmosomes. One of the desmosomes is still intact (red oval circle), while the other is disrupted (red asterisk). The big gold particles detect DSG1. The small gold particles detect Px4-3. **(A.II)** Picture showing indirect immunofluorescence microscopy staining for antibodies against DSG1. **(B.I)** Electron micrograph showing disrupted desmosomes. The gold particles (red arrows) mark DSG3. **(B.II)** Indirect immunofluorescence microscopy with intercellular staining by antibodies to DSG3. **(C.I)** Electron micrograph showing a stretched desmosome. Gold particles (red arrows) detect Px4-3 (anti-DSG1/3 scFv) within the desmosome. **(C.II)** Direct immunofluorescence microscopy against Px4-3 (the blister is marked by a red asterisk). Left column: Electron micrographs (100,000-fold; scale bars 0.2 μm), right column: Micrographs (200-fold; scale bars 100 μm).

**FIGURE 2:**
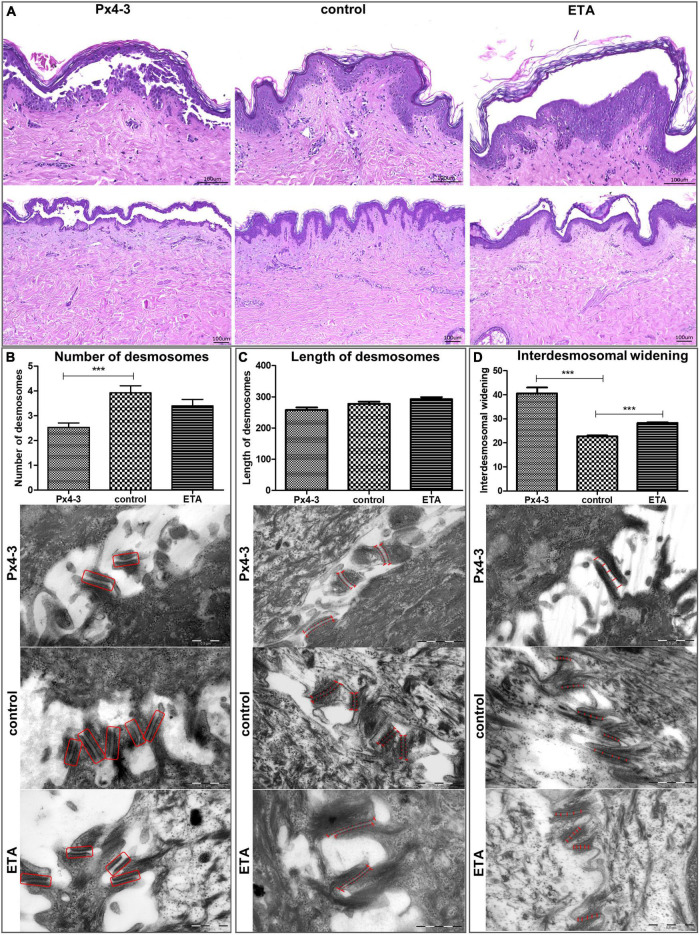
Desmosomal number, length, and interdesmosomal widening in the human skin organ culture model for pemphigus. **(A)** Light micrographs of hematoxylin and eosin stainings (top Figures 200-fold, scale bar 0.5 μm, *N* = 5, figures below: 100-fold, scale bar 1 μm, *N* = 5) of human skin organ culture specimens after injection of Px4-3, normal human IgG (negative control) or ETA (positive control and reference). **(B)** Electron micrographs showing the number of desmosomes in the three different conditions. Px4-3 injected has a smaller number of desmosomes compared to the control. **(C)** Electron micrographs showing the length of desmosomes. Px4-3 and ETA injected do not differ compared to the control. **(D)** Electron micrographs showing interdesmosomal widening. Px4-3 injected and ETA injected show larger interdesmosomal space than the control (80,000-fold, scale bar 0.5 μm, *N* = 5). ***p < 0.001.

The local ethics committee approved this study (06-109), realized according to the Declaration of Helsinki. We performed the HSOC following established protocols (15). In brief, human skin samples were obtained from donors without a history of skin diseases and were injected intradermally with either 50 μL of Px4-3 (60 μg) or ETA (100 ng). Normal human IgG was used as a control. Each experimental condition was evaluated in abdominal skin biopsies from five different donors. After 24 h of incubation, we processed the samples for histological and ultrastructural electron microscopy analyses, as described (15, 19). To demonstrate the Px4-3 binding at the desmosomes and to confirm epidermal split formation, we performed immunogold-labeling (Figure 1) and hematoxylin-eosin staining (Figure 2). For transmission electron microscopy (TEM) studies, fixation was performed using paraformaldehyde/piperazine-N-N′ bis (20-ethanol sulfonic acid) 5%, followed by Monti Graziadei or polyvinylpyrolidine-saccharose. After slide processing, we took 10 to 13 TEM pictures magnified 80,000-fold for each condition and skin. We used the “iTEM” software to count the desmosomes, measure their length and interdesmosomal widening. For Gaussian-distributed data, we used one-way ANOVA and Bonferroni post-test; for non-Gaussian-distributed data, Kruskal–Wallis and Dunn’s post-test. *P*-values lower than 0.05 were considered significant. Supplementary material presents more detailed information.

## Results

We evaluated the DSG1 and DSG3 expression to characterize the tissue of the model. Both were detected with immunogold-labeling and immunofluorescence staining in intact and/or disrupted desmosomes (Figures 1A,B). Immunogold-labeling and immunofluorescence staining demonstrated precisely Px4-3 binding to DSG1 and DSG3 in the PV model (Figure 1C). As expected, Px4-3 or ETA-injected skin specimens developed split formation at the corresponding intraepidermal layers, suprabasal stratum after Px4-3 injection and stratum granulosum after ETA-injection (Figure 2A). Px4-3 but not ETA injection reduced the desmosome number compared to control skin (Figure 2B). Interdesmosomal widening was seen in both Px4-3 or ETA injected skin and were significantly higher in both groups compared to control skin (Figure 2D). The length of the desmosomes does not differ between Px4-3 and ETA injected and controls (Figure 2C).

## Discussion

Consistent with our findings, Sokol et al. reported a reduced number of desmosomes in skin biopsies of PV patients (20). Egu et al. observed the same in human skin injected with IgG from patients with mucocutaneous PV (DSG1 and DSG3 autoantibodies) (10). In the same study, similar with our finds, higher interdesmosomal widening was observed (10). In contrast to our HSOC model, these studies reported reduced desmosomal size, as well in a recent study (21). Van der Wier et al. reported a reduced number of desmosomes in Nikolsky-positive PF biopsies but no difference in Nikolsky-negative PF biopsies compared to normal skin (22, 23). In line with our findings, they did not find changes in the desmosomal sizes in mucosal-dominant PV and Nikolsky-negative mucocutaneous PV biopsies compared to controls (23). Thus, the ultrastructural morphological features of desmosomes in the pemphigus HSOC model are similar to patient lesions (20). However, it does not show all of the ultrastructural hallmarks compared to the physiological human skin. Taken together, although desmosomal lengths differ between our model and PV/PF biopsies and other pemphigus models (10, 20, 24), we observed a lower number of desmosomes (10, 20) and a higher interdesmosomal widening across our model, as well as in patient skin biopsies (10). The usage of Px4-3 instead of PV-IgG, which contains antibodies against other adhesion molecules, cell membrane receptors, and mitochondrial antigens (25, 26), may explain the absence of observations of reduced desmosomal length in our model. The small amount of Px4-3 binding in the desmosomes may also indicate that pathogenic effects are caused by disturbed desmosome assembly or signaling induced by extradesmosomal desmogleins 1 and 3 (1, 27).

We conclude that, although our model has only bi-specific anti-DSG1/DSG3 scFv, some ultrastructural hallmarks of desmosome morphology following Px4-3 binding are reproduced in our HSOC model, reflecting the lesional skin of pemphigus patients. The reproducibility of the HSOC pemphigus model makes it an attractive tool to unravel novel therapeutic targets and evaluate new treatments targeting pemphigus pathology.

## Data availability statement

The original contributions presented in this study are included in the article/Supplementary material, further inquiries can be directed to the corresponding author.

## Ethics statement

The studies involving human participants were reviewed and approved by Ethics Committee of the University of Lübeck (06-109). The Ethics Committee waived the requirement of written informed consent for participation.

## Author contributions

JH and MK contributed to the conception of the work. JH, UR, and VB-BH designed the study. JH, CH, RL, and DZ provided the infrastructure and material for the HSOC. MK made available the infrastructure and material for the electron microscopy. UR and VB-BH performed the HSOC. UR collected the data. VB-BH did the statistical analysis. VB-BH, UR, JH, and AB drafted the manuscript. All authors critically evaluated the data, revised the work for intellectual content, revised the manuscript, and approved its final version.
