# Supplementary material for: Electron microscopy of desmosomal structures in the pemphigus human skin organ culture model
Source: Front Med (Lausanne). 2022 Nov 14;9:997387. doi: 10.3389/fmed.2022.997387 (PMC9701718; doi:10.3389/fmed.2022.997387)
Supplement: Supplementary file 1 [file Data_Sheet_1.docx]

Supplementary Material

# Supplementary material and methods

## Tissue collection

Skin donators of elective surgery from the practice of Dr. Valina in Lübeck, of Dr. Schlichter in Bremen and from the surgery of the University Clinic Schleswig-Holstein, Campus Lübeck were informed about the opportunity to donate skin for experimental work approved by the ethics committee of the University of Lübeck (06-109). Five skin donators voluntarily agreed to participate in the study and gave their informed consent.

## Human skin organ culture

From five different donors, for each experimental condition, we sampled two pieces of skin. We injected 50 µL of anti-desmoglein (DSG) 1/3 single-chain variable fragment (scFv). The scFv is a monoclonal antibody directed against DSG1 and 3, termed Px4-3, able to induce acantholysis (Hammers *et al*., 2015; Payne *et al*., 2005). The usage of Px4-3 was established in the Lübeck Institute of Experimental Dermatology (LIED). As a reference and positive control, we injected 50 µL of exfoliative toxin A (ETA) (Toxin Technology, Sarasota, USA), a toxin of the bacterium Staphylococcus aureus. As a negative control, we administrated 50 µL of normal human IgG. Intravenous immunoglobulin G (IVIG) (Biotest Pharma GmbH, Dreieich, Germany) was used as the source of normal human IgG. The skin specimens were harvested after incubation for 24 hours at 37 °C and 5 % CO2 for 24 hours. We used the first skin sample for hematoxylin-eosin (HE) staining and three different immunofluorescence stainings directed against DSG1, DSG3 and Px4-3. We used the second skin sample from each experimental condition for four 2 mm biopsy punches. After punching, the biopsies were transferred directly into 5 % paraformaldehyde (PFA)/ piperazine-N,N′-bis(2-ethanesulfonic acid)(PIPES) for fixation (PFA, distilled water, sodium hydroxide solution 1 mM, PIPES). They remained there for two to four hours (hrs). After lying in PFA/PIPES, we washed the punches in 0.14 M phosphate buffered saline (PBS) (sodium chloride, monopotassium phosphate, potassium chloride, disodium hydrogen phosphate). We stored two of the four punches in glutaraldehyde in cacodylate buffer for later araldit-embedding for standard transmission electron microscope (TEM) pictures. We put the other two punches in polyvinylpyrolidine (PVP)-saccharose for later immunogold-labeling (polyvinylpyrolidine, sucrose, PBS, sodium).

## Sample preparation for electron microscopy

The two skin punches that we stored in PVP-saccharose were used for the immunogold-labeling. The other two punches of each sample we put into glutaraldehyde in cacodylate buffer (instead of PVP-saccharose). After the storage in glutaraldehyde in cacodylate buffer, the skin punches were embedded in araldit.

## Araldit-embedding for standard electron microscopy

For the araldit-embedding, the biopsies first had to be fixed in glutaraldehyde 2.5 % for a minimum of 2 h. We washed the skin three times, every 5 min, in cacodylate buffer (pH = 7.2 – 7.4; 0.1 M). After that, we performed osmium fixation in 1 – 2 % OsO4 (in cacodylate buffer) for 2 h, followed by another three washing steps each for 10 min in cacodylate buffer. Dehydration is accomplished with an ethanol-series with increasing concentrations - twice 10 min starting with 30 up to 100 % ethanol, followed by incubation in 70 % ethanol with 1 % uranylacetate for a minimum of 1 h and protected from UV-light, concluding finally with 70 % ethanol overnight at 4 °C. After the dehydration, the punches were plunged twice into propylenoxid for 30 min and then transferred to propylenoxid-resin mixtures and a pure resin-mixture. After embedding in araldit, we stored the samples at room temperature (RT) until further usage.

We cut the samples with an ultramicrotome (Ultracut E, Reichert-Jung, Leica Mikrosysteme Vertrieb GmbH®, Wetzlar, Germany). After that, we included the sliced samples into Leica EM AC20 Automatic Contrasting Instrument (Leica Mikrosysteme Vertrieb GmbH®, Wetzlar, Germany). We took 10 to 13 pictures for each experimental model at the TEM (JEOL JEM-1011, JEOL®, Tokyo, Japan). These pictures were used for our analyses of the desmosomes.

For this, we used the program iTEM (www.soft-imaging.net). First of all, we measured the area of the TEM pictures. We took all of the micrographs for the analyses of the desmosomes in 80,000 magnification. First, we counted the desmosomes in every micrograph. For each of these counted desmosomes, we measured desmosomal length and interdesmosomal widening. For determining the length of the desmosomes, we measured both desmosomal plaques of each desmosome, using the feature "polyline”. After that, we calculated the average of the two values for each desmosome. We measured the distance between the desmosomal plaques by choosing five points. Finally, we calculated the average of the five values for each desmosomal distance.

## Immunogold-labeling in electron microscopy

We included the punches from the PVP-saccharose into a medium for thawing onto pins (Leica Mikrosysteme Vertrieb GmbH®, Wetzlar, Germany). We cut the skin biopsies and put the pins underneath the stereo magnifier using a clamp. Using a loop, the slide got wetted homogeneously with a thawing medium. The skin biopsies were freed from PVP-saccharose with filter paper. After that, we placed them onto the pins. Finally, we stored the pins with the skin punches in liquid nitrogen until further usage. We cut them with the cryo-ultramicrotome (Leica EM FCS, Leica Mikrosysteme Vertrieb GmbH®, Wetzlar, Germany) to produce 60 to 80 nm thick sections. For cutting, we used three different temperatures within the ultra-cryo. The block temperature was set between -110 °C to -120 °C, the temperature of the diamond knife, between -100 °C to -115 °C and the temperature of the chamber, -130 °C. We gently put the sections onto grids (Formvar covered, mesh = 100).

The immunogold-labeling followed the cutting process. First, we incubated the sections for 15 mins with 10 % fetal calf serum (FCS) (1 drop to block the unspecific antigens), then 45 min with the primary antibody [for DSG1: mouse anti-human desmoglein-1 (Origene®, Rockville, United States of America, # AM26377PU-N), for DSG3: mouse anti-human desmoglein-3 (Origene®, Rockville, United States of America, # SM2037PS), for Px4-3: rat anti-HA mAb high affinity 0,1 µg/µL (Roche®, Basel, Switzerland); each of the antibodies was diluted in 5 % FCS]. After this, 15 min of washing with 0.14 M PBS (5 drops) followed. Then, 45 min incubation with the secondary antibody [6 nm Colloidal Gold-AffiniPure Goat Anti-Rat IgG (Jackson Immuno Research®, West Grove United States of America, # 112-195-167), diluted in 5 % FCS/ 12 nm Colloidal Gold-AffiniPure Goat Anti-Mouse IgG (Jackson Immuno Research®, West Grove United States of America, # 115-205-146), diluted in 5 % FCS]. After the incubation, 30 min of washing was done in 0.14 M PBS (6 drops) and 5 min of washing with ddH2O (3 drops). Then, we put the sections onto ice with 100 µL uranylacetate (4 %) and we poured 900 µL of methylcellulose (2 %) on top. Sections were dried afterwards. After immunogold-labeling, we took the pictures with the routine transmission electron microscopy (JEOL JEM-1011, JEOL, Tokyo, Japan).

## Hematoxylin and eosin staining

After embedding into paraffin, we cut 4 µm thick sections from each paraffin block. We deparaffinized the dried sections in a xylene bath and dehydrated them in an increasing ethanol concentration series. Afterward, we dipped the slides into distilled water, then into papanicoulos-hematoxylin for 5 min. After the hematoxylin, we rinsed the slides in water, put them into ammonia water, rinsed them in the water again and then dipped them into distilled water. The eosin bath followed and another increasing ethanol concentration series as well, as xylene baths. In the end, the coverslipping machine coverslipped the sections.

## Immunofluorescence staining for desmogleins

We cut the skin into 6 µm sections. These were used for immunofluorescence stainings directed against DSG1 and 3. The sections first thawed for 10 min. After fixation in acetone for 20 min, we encircled each section using a fat pen (DAKO®, Hamburg, Germany, # S2002). Two washing steps in PBS followed, each for 5 min. Preincubation with 10 % goat normal serum (GNS) for 20 min followed. The primary antibody of each staining [for DSG1: (Origene®, Rockville, United States of America, # AM26377PU-N) mouse anti-human DSG1, for DSG3: (Origene®, Rockville, United States of America, # SM2037PS) staining the primary antibody mouse anti-human DSG 3) and 2 % GNS was added diluted in PBS (1:100).] We stored the humid chamber including the sections overnight in the fridge at 4 °C. Three washing steps in PBS followed, each for 5 min. Then, we performed the incubation with the secondary antibody [for DSG1: CyTM 3-conjugated AffiniPure goat anti-mouse IgG (Jackson Immuno Research®, West Grove United States of America, # 115-165-146), for DSG3: Goat Anti-Mouse IgG (H + L) Alexa Fluor 488 (Jackson Immuno Research®, West Grove United States of America, # A11029); dilution: 1:200 in PBS] for 45 min. We washed the sections three times in PBS, each for 5 min. We covered the slides with 4,6-diamidine-2-phenylindole (DAPI)-fluoromount-G and coverslips, dried for one hour and stored them at - 20° C.

## Immunofluorescence staining for Px4-3

We prepared washing and blocking buffers. For the washing buffer, we added 100 mL TBS, 500 µL 2 M CaCl2, 900 mL NanoPure ddH2O and 500 µL of Tween 20. The blocking buffer consists 2.5 g of bovine albumin (BSA), which was filled into a falcon tube and filled up with washing buffer to a volume of 250 mL. We filtered the mixture before storing it at 4 °C. The sections were thawed for 3 to 5 min. We encircled each section using a fat pen. We washed the sections twice with washing buffer for 5 min, then blocked them with the blocking buffer for 15 min and afterwards incubated them with the secondary antibody [Rat Anti- HA High-Affinity Antibody monoclonal (Roche®, Basel, Switzerland, # 11867423001), dilution 1:100 in blocking buffer] for 1 h. After three times washing, we added the tertiary antibody [Goat Alexa Fluor 594 Anti-Rat IgG (Life Technologies®, Carlsbad, United States of America, # A11007), dilution 1:200 in blocking buffer)] for 1 h. Afterwards, we washed the sections three times in washing buffer, each for 5 min. We covered the slides with DAPI-fluoromount-G and coverslips, dried them for one hour and stored them at - 20 °C.

## Microscopical analyses

While using the Keyence BZ 9000 (Keyence®, Osaka, Japan) the HE-stained slides were observed with 100 magnification. We took pictures along the whole epidermis.

## Semi-quantitative histomorphometry

Every picture taken described a visual field of the skin. For every visual field, we measured the length of the epidermis using the program ImageJ (https://fiji.sc). We measured the whole epidermis length as well as the split formation within the visual fields. Two hundred µm of both skin edges were taken out of the evaluation to avoid cutting artifacts.

# Supplementary Table 1. Number of desmosomes, length of desmosomes and interdesmosomal widening in five Px4-3-injected HSOCs, five controls and five ETA-injected HSOCs.

| **Picture number** | **Number of desm.** | **Desm. number** | **Length** | **Int. wid.** | **Picture number** | **Number of desm.** | **Desm. number** | **Length** | **Int. wid.** | **Picture number** | **Number of desm.** | **Desm. number** | **Length** | **Int. wid.** |
| --- | --- | --- | --- | --- | --- | --- | --- | --- | --- | --- | --- | --- | --- | --- |
| **Px4-3**  **HSOC 1** |  |  |  |  | **Control HSOC 1** |  |  |  |  | **ETA**  **HSOC 1** |  |  |  |  |
| **1** | 2 | 1 | 338.89 | 22.83 | **1** | 9 | 1 | 307.28 | 14.76 | **1** | 2 | 1 | 203.03 | 25.99 |
|  |  | 2 | 328.74 | 26.06 |  |  | 2 | 259.55 | 15.24 |  |  | 2 | 290.13 | 24.12 |
|  |  | . | . | . |  |  | 3 | 146.98 | 22.05 |  |  | . | . | . |
|  |  | . | . | . |  |  | 4 | 256.44 | 17 |  |  | . | . | . |
|  |  | . | . | . |  |  | 5 | 247.76 | 16.75 |  |  | . | . | . |
|  |  | . | . | . |  |  | 6 | 279.58 | 24.36 |  |  | . | . | . |
|  |  | . | . | . |  |  | 7 | 317.99 | 21.76 |  |  | . | . | . |
|  |  | . | . | . |  |  | 8 | 352.24 | 24.94 |  |  | . | . | . |
|  |  | . | . | . |  |  | 9 | 237.93 | 25.84 |  |  | . | . | . |
| **2** | 4 | 1 | 188.6 | 30.26 | **2** | 6 | 1 | 252.2 | 25.09 | **2** | 3 | 1 | 327.45 | 28.85 |
|  |  | 2 | 164.96 | 24.77 |  |  | 2 | 328.41 | 21.53 |  |  | 2 | 470 | 26.37 |
|  |  | 3 | 292.36 | 30.09 |  |  | 3 | 152.14 | 24.64 |  |  | 3 | 275.28 | 23.5 |
|  |  | 4 | 281.18 | 33.02 |  |  | 4 | 231.48 | 13.9 |  |  |  | . | . |
|  |  |  | . | . |  |  | 5 | 296.65 | 23.24 |  |  |  | . | . |
|  |  |  | . | . |  |  | 6 | 214.12 | 29.95 |  |  |  | . | . |
| **3** | 5 | 1 | 313.98 | 26.13 | **3** | 2 | 1 | 385.67 | 19.3 | **3** | 3 | 1 | 228.38 | 23.57 |
|  |  | 2 | 104.66 | 31.7 |  |  | 2 | 355.52 | 24.21 |  |  | 2 | 237.11 | 21.26 |
|  |  | 3 | 210.98 | 18.82 |  |  |  | . | . |  |  | 3 | 333.23 | 25.42 |
|  |  | 4 | 396.1 | 116.4 |  |  |  | . | . |  |  |  | . | . |
|  |  | 5 | 223.49 | 31.32 |  |  |  | . | . |  |  |  | . | . |
| **4** | 3 | 1 | 176.42 | 31.08 | **4** | 3 | 1 | 309.43 | 21.55 | **4** | 3 | 1 | 283.79 | 24.08 |
|  |  | 2 | 103.7 | 27.27 |  |  | 2 | 111.52 | 23.68 |  |  | 2 | 263.86 | 23.56 |
|  |  | 3 | 174.04 | 50.41 |  |  | 3 | 441.45 | 23.76 |  |  | 3 | 498.62 | 25.09 |
| **5** | 2 | 1 | 346.47 | 23.11 | **5** | 3 | 1 | 328.72 | 22.46 | **5** | 6 | 1 | 205.29 | 24.41 |
|  |  | 2 | 119.63 | 22.78 |  |  | 2 | 213.6 | 24.74 |  |  | 2 | 224.62 | 25.86 |
|  |  |  | . | . |  |  | 3 | 378.23 | 22.3 |  |  | 3 | 148.88 | 21.26 |
|  |  |  | . | . |  |  |  | . | . |  |  | 4 | 195.98 | 29.74 |
|  |  |  | . | . |  |  |  | . | . |  |  | 5 | 217.2 | 26.39 |
|  |  |  | . | . |  |  |  | . | . |  |  | 6 | 120.96 | 31.36 |
| **6** | 3 | 1 | 68 | 26.75 | **6** | 2 | 1 | 593.44 | 23.22 | **6** | 4 | 1 | 431.46 | 25.45 |
|  |  | 2 | 113.38 | 27.53 |  |  | 2 | 378.04 | 22.03 |  |  | 2 | 309.87 | 25.3 |
|  |  | 3 | 212.66 | 27.96 |  |  |  | . | . |  |  | 3 | 236.2 | 22.87 |
|  |  |  | . | . |  |  |  | . | . |  |  | 4 | 293.83 | 24.33 |
| **7** | 1 | 1 | 218.14 | 35.19 | **7** | 2 | 1 | 196.25 | 23.03 | **7** | 2 | 1 | 254.28 | 29.47 |
|  |  |  | . | . |  |  | 2 | 320.91 | 22.03 |  |  | 2 | 152.19 | 26.7 |
| **8** | 1 | 1 | 206.79 | 29.34 | **8** | 8 | 1 | 331.52 | 24.14 | **8** | 3 | 1 | 263.49 | 22.41 |
|  |  |  | . | . |  |  | 2 | 362.73 | 18.43 |  |  | 2 | 266.02 | 23.73 |
|  |  |  | . | . |  |  | 3 | 255.43 | 22.93 |  |  | 3 | 200.08 | 17.45 |
|  |  |  | . | . |  |  | 4 | 173.91 | 20.54 |  |  |  | . | . |
|  |  |  | . | . |  |  | 5 | 251.58 | 23.2 |  |  |  | . | . |
|  |  |  | . | . |  |  | 6 | 280.88 | 18.95 |  |  |  | . | . |
|  |  |  | . | . |  |  | 7 | 158.96 | 23.43 |  |  |  | . | . |
|  |  |  | . | . |  |  | 8 | 230.15 | 17.45 |  |  |  | . | . |
| **9** | 4 | 1 | 166.97 | 31.75 | **9** | 9 | 1 | 282.8 | 23.14 | **9** | 3 | 1 | 273.7 | 20.71 |
|  |  | 2 | 145.32 | 37.05 |  |  | 2 | 401.87 | 20.89 |  |  | 2 | 267.62 | 21.8 |
|  |  | 3 | 160.87 | 31.97 |  |  | 3 | 183.35 | 20.99 |  |  | 3 | 493.49 | 22.92 |
|  |  | 4 | 149.59 | 31.09 |  |  | 4 | 233.7 | 20.54 |  |  |  | . | . |
|  |  |  | . | . |  |  | 5 | 255.09 | 25.2 |  |  |  | . | . |
|  |  |  | . | . |  |  | 6 | 191.14 | 16.95 |  |  |  | . | . |
|  |  |  | . | . |  |  | 7 | 299.59 | 23.42 |  |  |  | . | . |
|  |  |  | . | . |  |  | 8 | 154.58 | 17.55 |  |  |  | . | . |
|  |  |  | . | . |  |  | 9 | 161.82 | 22.48 |  |  |  | . | . |
| **10** | 4 | 1 | 198.71 | 103.36 | **10** | 3 | 1 | 281.3 | 25.03 | **10** | 3 | 1 | 342.11 | 27.79 |
|  |  | 2 | 179.81 | 26.2 |  |  | 2 | 252.05 | 23.33 |  |  | 2 | 332.5 | 22.67 |
|  |  | 3 | 386.58 | 27.92 |  |  | 3 | 91.33 | 21.91 |  |  | 3 | 335.5 | 27.31 |
|  |  | 4 | 208.17 | 31.23 |  |  |  | . | . |  |  |  | . | . |
|  |  |  |  |  | **11** | 3 | 1 | 398.81 | 29.28 | **11** | 2 | 1 | 332.33 | 26.98 |
|  |  |  |  |  |  |  | 2 | 366.19 | 24.69 |  |  | 2 | 259.07 | 32.71 |
|  |  |  |  |  |  |  | 3 | 288.18 | 25.06 |  |  |  | . | . |
|  |  |  |  |  | **12** | 2 |  | 295.79 | 24.59 | **12** | 3 |  | 434.46 | 26.12 |
|  |  |  |  |  |  |  |  | 551.36 | 28.22 |  |  |  | 238.14 | 27.67 |
|  |  |  |  |  |  |  |  | . | . |  |  |  | 489.61 | 25.56 |
|  |  |  |  |  | **13** | 1 |  | 376.68 | 31.17 | **13** | 2 |  | 380.81 | 27.09 |
|  |  |  |  |  |  |  |  | . | . |  |  |  | 375.44 | 33.43 |
| **Px4-3 HSOC 2** |  |  |  |  | **Control HSOC 2** |  |  |  |  | **ETA**  **HSOC 2** |  |  |  |  |
| **1** | 3 | 1 | 303.91 | 83.22 | **1** | 2 | 1 | 251.38 | 20.07 | **1** | 3 | 1 | 379.45 | 29.3 |
|  |  | 2 | 261.68 | 30.25 |  |  | 2 | 323.85 | 21.53 |  |  | 2 | 347.07 | 26.45 |
|  |  | 3 | 166.24 | 25.12 |  |  |  | . | . |  |  | 3 | 178.21 | 27.78 |
| **2** | 4 | 1 | 338.09 | 23.99 | **2** | 8 | 1 | 165.88 | 29.62 | **2** | 5 | 1 | 336.01 | 30.79 |
|  |  | 2 | 249.78 | 23.97 |  |  | 2 | 196.65 | 20.79 |  |  | 2 | 264.18 | 24.3 |
|  |  | 3 | 260.75 | 26.95 |  |  | 3 | 215.44 | 25.06 |  |  | 3 | 265.96 | 32.66 |
|  |  | 4 | 126.87 | 25.12 |  |  | 4 | 256.99 | 22.61 |  |  | 4 | 210.32 | 27.37 |
|  |  |  | . | . |  |  | 5 | 205.58 | 27.3 |  |  | 5 | 454.65 | 30.52 |
|  |  |  | . | . |  |  | 6 | 191.65 | 23.14 |  |  |  | . | . |
|  |  |  | . | . |  |  | 7 | 177.65 | 22.11 |  |  |  | . | . |
|  |  |  | . | . |  |  | 8 | 343.88 | 23.52 |  |  |  | . | . |
| **3** | 3 | 1 | 156.34 | 26.92 | **3** | 4 | 1 | 116.39 | 23.28 | **3** | 3 | 1 | 154.79 | 37.15 |
|  |  | 2 | 170.25 | 40.22 |  |  | 2 | 167.75 | 20.05 |  |  | 2 | 168.69 | 39.93 |
|  |  | 3 | 259.08 | 111.3 |  |  | 3 | 224.76 | 23.64 |  |  | 3 | 435.34 | 24.14 |
|  |  |  | . | . |  |  | 4 | 581.87 | 23.58 |  |  |  | . | . |
| **4** | 4 | 1 | 236.45 | 42.69 | **4** | 2 | 1 | 167.11 | 20.25 | **4** | 2 | 1 | 227.98 | 34.29 |
|  |  | 2 | 231.55 | 43.43 |  |  | 2 | 183.74 | 16.64 |  |  | 2 | 223.09 | 31.47 |
|  |  | 3 | 309.82 | 26.26 |  |  |  | . | . |  |  |  | . | . |
|  |  | 4 | 199.78 | 70.44 |  |  |  | . | . |  |  |  | . | . |
| **5** | 3 | 1 | 131.89 | 24.46 | **5** | 3 | 1 | 130.88 | 18.56 | **5** | 3 | 1 | 305.28 | 30.94 |
|  |  | 2 | 274.67 | 27.01 |  |  | 2 | 277.26 | 22.8 |  |  | 2 | 363.98 | 36.84 |
|  |  | 3 | 188.69 | 30.84 |  |  | 3 | 288.8 | 23.82 |  |  | 3 | 233.01 | 31.5 |
| **6** | 2 | 1 | 378.83 | 38.67 | **6** | 1 | 1 | 312.86 | 13.38 | **6** | 1 | 1 | 640.54 | 34.56 |
|  |  | 2 | 217.28 | 29.86 |  |  |  | . | . |  |  |  | . | . |
| **7** | 1 | 1 | 372.7 | 30.92 | **7** | 10 | 1 | 181.95 | 20.63 | **7** | 3 | 1 | 459.82 | 35.64 |
|  |  |  | . | . |  |  | 2 | 279.6 | 22.36 |  |  | 2 | 481.09 | 21.16 |
|  |  |  | . | . |  |  | 3 | 336.1 | 20.79 |  |  | 3 | 293.1 | 22.17 |
|  |  |  | . | . |  |  | 4 | 301.53 | 23.32 |  |  |  | . | . |
|  |  |  | . | . |  |  | 5 | 275.9 | 19.75 |  |  |  | . | . |
|  |  |  | . | . |  |  | 6 | 279.46 | 20.28 |  |  |  | . | . |
|  |  |  | . | . |  |  | 7 | 266.74 | 19.8 |  |  |  | . | . |
|  |  |  | . | . |  |  | 8 | 252.67 | 19.44 |  |  |  | . | . |
|  |  |  | . | . |  |  | 9 | 271.25 | 24.44 |  |  |  | . | . |
|  |  |  | . | . |  |  | 10 | 266.74 | 20.33 |  |  |  | . | . |
| **8** | 3 | 1 | 167.53 | 24.6 | **8** | 4 | 1 | 252.67 | 27.57 | **8** | 2 | 1 | 232.75 | 32.67 |
|  |  | 2 | 331.1 | 31.27 |  |  | 2 | 271.25 | 17.04 |  |  | 2 | 230.74 | 28.53 |
|  |  | 3 | 160.22 | 28.43 |  |  | 3 | 125.39 | 17.31 |  |  |  | . | . |
|  |  |  | . | . |  |  | 4 | 223.38 | 22.28 |  |  |  | . | . |
| **9** | 3 | 1 | 235.44 | 35.85 | **9** | 2 | 1 | 138.1 | 21.86 | **9** | 5 | 1 | 275.9 | 26.83 |
|  |  | 2 | 223.23 | 42.84 |  |  | 2 | 304.91 | 21.94 |  |  | 2 | 266.18 | 23.39 |
|  |  | 3 | 107.38 | 28.07 |  |  |  | . | . |  |  | 3 | 215.54 | 21.9 |
|  |  |  | . | . |  |  |  | . | . |  |  | 4 | 134.91 | 21.43 |
|  |  |  | . | . |  |  |  | . | . |  |  | 5 | 199.14 | 25.32 |
| **10** | 3 | 1 | 165.44 | 29.37 | **10** | 2 | 1 | 160.26 | 21.75 | **10** | 2 | 1 | 371.99 | 30.63 |
|  |  | 2 | 266.66 | 29 |  |  | 2 | 356.96 | 18.33 |  |  | 2 | 235.07 | 28.49 |
|  |  | 3 | 176.52 | 32.99 |  |  |  | . | . |  |  |  | . | . |
| **11** | 1 | 1 | 188.15 | 30.39 | **11** | 2 | 1 | 117.32 | 21.16 | **11** | 4 | 1 | 309.38 | 26.1 |
|  |  |  | . | . |  |  | 2 | 222.76 | 24.78 |  |  | 2 | 85.43 | 18.66 |
|  |  |  | . | . |  |  |  | . | . |  |  | 3 | 354.38 | 23.99 |
|  |  |  | . | . |  |  |  | . | . |  |  | 4 | 204.54 | 20.17 |
|  |  |  |  |  | **12** | 5 | 1 | 296.13 | 25.83 | **12** | 3 | 1 | 220.57 | 25.21 |
|  |  |  |  |  |  |  | 2 | 240.62 | 32.86 |  |  | 2 | 306.09 | 25.72 |
|  |  |  |  |  |  |  | 3 | 264.39 | 23.5 |  |  | 3 | 149.54 | 33.76 |
|  |  |  |  |  |  |  | 4 | 275.5 | 25.57 |  |  |  | . | . |
|  |  |  |  |  |  |  | 5 | 209.5 | 30.64 |  |  |  | . | . |
|  |  |  |  |  | **13** | 3 | 1 | 249.49 | 22.76 | **13** | 3 | 1 | 179.61 | 31.26 |
|  |  |  |  |  |  |  | 2 | 428.62 | 20.75 |  |  | 2 | 425.15 | 27.53 |
|  |  |  |  |  |  |  | 3 | 295.36 | 20.65 |  |  | 3 | 178.44 | 33.58 |
|  |  |  |  |  |  |  |  |  |  | **14** | 1 | 1 | 298.94 | 25.3 |
| **Px4-3**  **HSOC 3** |  |  |  |  | **Control HSOC 3** |  |  |  |  | **ETA**  **HSOC 3** |  |  |  |  |
| **1** | 3 | 1 | 153.76 | 43.4 | **1** | 5 | 1 | 123.54 | 32.39 | **1** | 1 | 1 | 228.31 | 31.04 |
|  |  | 2 | 165.91 | 25.81 |  |  | 2 | 320.87 | 28.07 |  |  |  | . | . |
|  |  | 3 | 233.96 | 240.9 |  |  | 3 | 276.3 | 25.59 |  |  |  | . | . |
|  |  |  | . | . |  |  | 4 | 280.73 | 17.06 |  |  |  | . | . |
|  |  |  | . | . |  |  | 5 | 188 | 20.47 |  |  |  | . | . |
| **2** | 1 | 1 | 261.95 | 30.5 | **2** | 1 | 1 | 333.79 | 24.98 | **2** | 5 | 1 | 247.05 | 28.75 |
|  |  |  | . | . |  |  |  | . | . |  |  | 2 | 524.96 | 26.65 |
|  |  |  | . | . |  |  |  | . | . |  |  | 3 | 280.07 | 31.81 |
|  |  |  | . | . |  |  |  | . | . |  |  | 4 | 303.47 | 34.1 |
|  |  |  | . | . |  |  |  | . | . |  |  | 5 | 460.07 | 25.3 |
| **3** | 1 | 1 | 309.6 | 61.85 | **3** | 2 | 1 | 325.55 | 26.3 | **3** | 2 | 1 | 313.46 | 26.76 |
|  |  |  | . | . |  |  | 2 | 383.8 | 16.36 |  |  | 2 | 318.26 | 26.36 |
| **4** | 1 | 1 | 358.39 | 36.54 | **4** | 3 | 1 | 184.09 | 22.77 | **4** | 3 | 1 | 277.45 | 24.27 |
|  |  |  | . | . |  |  | 2 | 291.29 | 22.82 |  |  | 2 | 231.48 | 22.25 |
|  |  |  | . | . |  |  | 3 | 239.88 | 20.81 |  |  | 3 | 284.52 | 38.49 |
| **5** | 3 | 1 | 283.09 | 36.7 | **5** | 2 | 1 | 164.9 | 23.37 | **5** | 2 | 1 | 286.8 | 31.31 |
|  |  | 2 | 511.78 | 31.4 |  |  | 2 | 259.2 | 26.44 |  |  | 2 | 331.74 | 31.15 |
|  |  | 3 | 266.63 | 35.72 |  |  |  | . | . |  |  |  | . | . |
| **6** | 1 | 1 | 273.9 | 23.8 | **6** | 6 | 1 | 421.48 | 29.33 | **6** | 6 | 1 | 264.63 | 31.68 |
|  |  |  | . | . |  |  | 2 | 403.33 | 18.32 |  |  | 2 | 178 | 23.67 |
|  |  |  | . | . |  |  | 3 | 173.89 | 14.48 |  |  | 3 | 280.73 | 41.28 |
|  |  |  | . | . |  |  | 4 | 296.77 | 18.62 |  |  | 4 | 222.73 | 29.82 |
|  |  |  | . | . |  |  | 5 | 280.68 | 18.41 |  |  | 5 | 240.63 | 28.16 |
|  |  |  | . | . |  |  | 6 | 244.53 | 18.13 |  |  | 6 | 218.29 | 23.95 |
| **7** | 2 | 1 | 362.54 | 37.54 | **7** | 7 | 1 | 258.95 | 25.64 | **7** | 4 | 1 | 224.59 | 26.6 |
|  |  | 2 | 197.65 | 31.43 |  |  | 2 | 200.91 | 20.54 |  |  | 2 | 212.77 | 18.1 |
|  |  |  | . | . |  |  | 3 | 179.36 | 17.25 |  |  | 3 | 268.23 | 22.05 |
|  |  |  | . | . |  |  | 4 | 229.29 | 17.65 |  |  | 4 | 236.44 | 34.55 |
|  |  |  | . | . |  |  | 5 | 254 | 20.18 |  |  |  | . | . |
|  |  |  | . | . |  |  | 6 | 140.1 | 23.24 |  |  |  | . | . |
|  |  |  | . | . |  |  | 7 | 268.72 | 10.44 |  |  |  | . | . |
| **8** | 2 | 1 | 397.98 | 30.48 | **8** | 8 | 1 | 274.75 | 16.03 | **8** | 4 | 1 | 211.44 | 20.66 |
|  |  | 2 | 276.47 | 26.31 |  |  | 2 | 150.84 | 14.89 |  |  | 2 | 250.91 | 30.17 |
|  |  |  | . | . |  |  | 3 | 222.19 | 12.45 |  |  | 3 | 364.75 | 18.74 |
|  |  |  | . | . |  |  | 4 | 181.7 | 27.66 |  |  | 4 | 338.42 | 37.18 |
|  |  |  | . | . |  |  | 5 | 255.31 | 16.57 |  |  |  | . | . |
|  |  |  | . | . |  |  | 6 | 206.57 | 17.42 |  |  |  | . | . |
|  |  |  | . | . |  |  | 7 | 340.05 | 20.15 |  |  |  | . | . |
|  |  |  | . | . |  |  | 8 | 230.77 | 19.08 |  |  |  | . | . |
| **9** | 1 | 1 | 309.5 | 31.81 | **9** | 5 | 1 | 170.6 | 20.18 | **9** | 2 | 1 | 354.8 | 31.13 |
|  |  |  | . | . |  |  | 2 | 287.65 | 20.06 |  |  | 2 | 404.69 | 26.82 |
|  |  |  | . | . |  |  | 3 | 333.66 | 28.42 |  |  |  | . | . |
|  |  |  | . | . |  |  | 4 | 247.11 | 21.45 |  |  |  | . | . |
|  |  |  | . | . |  |  | 5 | 560.01 | 21.25 |  |  |  | . | . |
| **10** | 2 | 1 | 226.4 | 26.13 | **10** | 3 | 1 | 249.23 | 23.82 | **10** | 3 | 1 | 226.45 | 23.05 |
|  |  | 2 | 431.94 | 26.27 |  |  | 2 | 308.64 | 22.44 |  |  | 2 | 530.34 | 26.6 |
|  |  |  |  |  |  |  | 3 | 229.63 | 17.23 |  |  | 3 | 336.54 | 33.88 |
| **11** | 3 | 1 | 245.01 | 28.68 | **11** | 4 | 1 | 253.46 | 20.99 | **11** | 11 | 1 | 357.26 | 34.62 |
|  |  | 2 | 198.95 | 23.4 |  |  | 2 | 278.1 | 17.61 |  |  | 2 | 331.83 | 27.18 |
|  |  | 3 | 233.52 | 85.38 |  |  | 3 | 318.93 | 22.26 |  |  | 3 | 311.11 | 30.59 |
|  |  |  | . | . |  |  | 4 | 470.84 | 23.48 |  |  | 4 | 278.91 | 28.25 |
|  |  |  | . | . |  |  |  | . | . |  |  | 5 | 220.13 | 25.36 |
|  |  |  | . | . |  |  |  | . | . |  |  | 6 | 226.93 | 28.63 |
|  |  |  | . | . |  |  |  | . | . |  |  | 7 | 295.09 | 21.88 |
|  |  |  | . | . |  |  |  | . | . |  |  | 8 | 325.43 | 28.48 |
|  |  |  | . | . |  |  |  | . | . |  |  | 9 | 350.02 | 22.1 |
|  |  |  | . | . |  |  |  | . | . |  |  | 10 | 262.25 | 28.55 |
|  |  |  | . | . |  |  |  | . | . |  |  | 11 | 220.28 | 25.26 |
| **12** | 2 | 1 | 470.51 | 135 | **12** | 4 | 1 | 276.01 | 18.03 | **12** | 3 | 1 | 323.97 | 28.94 |
|  |  | 2 | 235.11 | 33.47 |  |  | 2 | 442.61 | 22.16 |  |  | 2 | 580.74 | 23.77 |
|  |  |  | . | . |  |  | 3 | 490.19 | 24.45 |  |  | 3 | 350.94 | 24.38 |
|  |  |  | . | . |  |  | 4 | 442.78 | 23.26 |  |  |  | . | . |
| **13** | 6 | 1 | 516.26 | 25.05 |  |  |  |  |  | **13** | 5 | 1 | 194.02 | 25.88 |
|  |  | 2 | 227.86 | 24.8 |  |  |  |  |  |  |  | 2 | 290.77 | 21.6 |
|  |  | 3 | 302 | 23.43 |  |  |  |  |  |  |  | 3 | 232.07 | 28.03 |
|  |  | 4 | 180.19 | 28.35 |  |  |  |  |  |  |  | 4 | 281.95 | 28.79 |
|  |  | 5 | 131.17 | 32.88 |  |  |  |  |  |  |  | 5 | 197.26 | 25.88 |
|  |  | 6 | 259.16 | 29.94 |  |  |  |  |  |  |  | . | . | . |
| **14** | 8 | 1 | 187.31 | 37.58 |  |  |  |  |  |  |  |  |  |  |
|  |  | 2 | 139.85 | 31.05 |  |  |  |  |  |  |  |  |  |  |
|  |  | 3 | 296.87 | 26.93 |  |  |  |  |  |  |  |  |  |  |
|  |  | 4 | 258.92 | 24.46 |  |  |  |  |  |  |  |  |  |  |
|  |  | 5 | 179.12 | 82.91 |  |  |  |  |  |  |  |  |  |  |
|  |  | 6 | 266 | 23.85 |  |  |  |  |  |  |  |  |  |  |
|  |  | 7 | 229.62 | 34.74 |  |  |  |  |  |  |  |  |  |  |
|  |  | 8 | 138.41 | 35.74 |  |  |  |  |  |  |  |  |  |  |
| **15** | 1 | 1 | 486.39 | 25.81 |  |  |  |  |  |  |  |  |  |  |
| **Px4-3**  **HSOC 4** |  |  |  |  | **Control HSOC 4** |  |  |  |  | **ETA**  **HSOC 4** |  |  |  |  |
| **1** | 2 | 1 | 273.84 | 24.24 | **1** | 6 | 1 | 247.96 | 35.18 | **1** | 1 | 1 | 357.44 | 24.95 |
|  |  | 2 | 287.26 | 33.71 |  |  | 2 | 176.51 | 34.7 |  |  |  | . | . |
|  |  |  | . | . |  |  | 3 | 126.06 | 38.93 |  |  |  | . | . |
|  |  |  | . | . |  |  | 4 | 179.92 | 32.22 |  |  |  | . | . |
|  |  |  | . | . |  |  | 5 | 100.86 | 32.68 |  |  |  | . | . |
|  |  |  | . | . |  |  | 6 | 203.71 | 32.2 |  |  |  | . | . |
| **2** | 3 | 1 | 485.03 | 28.15 | **2** | 6 | 1 | 188.52 | 36.4 | **2** | 2 | 1 | 338.68 | 25.29 |
|  |  | 2 | 374.88 | 30.68 |  |  | 2 | 246.98 | 34.65 |  |  | 2 | 454.62 | 22.75 |
|  |  | 3 | 416.95 | 33.91 |  |  | 3 | 172.53 | 24.09 |  |  |  | . | . |
|  |  |  | . | . |  |  | 4 | 427.4 | 27.42 |  |  |  | . | . |
|  |  |  | . | . |  |  | 5 | 226.87 | 22.28 |  |  |  | . | . |
|  |  |  | . | . |  |  | 6 | 245.95 | 21.2 |  |  |  | . | . |
| **3** | 3 | 1 | 239.46 | 35.06 | **3** | 8 | 1 | 176.62 | 15.53 | **3** | 1 | 1 | 311.1 | 28.01 |
|  |  | 2 | 260.32 | 31.97 |  |  | 2 | 469.7 | 24.69 |  |  |  | . | . |
|  |  | 3 | 360.28 | 133.4 |  |  | 3 | 269.85 | 25.28 |  |  |  | . | . |
|  |  |  | . | . |  |  | 4 | 376.47 | 26.54 |  |  |  | . | . |
|  |  |  | . | . |  |  | 5 | 263.03 | 21.26 |  |  |  | . | . |
|  |  |  | . | . |  |  | 6 | 164.07 | 13.19 |  |  |  | . | . |
|  |  |  | . | . |  |  | 7 | 250.28 | 22.62 |  |  |  | . | . |
|  |  |  | . | . |  |  | 8 | 227.83 | 24.48 |  |  |  | . | . |
| **4** | 5 | 1 | 220.8 | 26.43 | **4** | 9 | 1 | 169.22 | 29.46 | **4** | 2 | 1 | 336.97 | 26.09 |
|  |  | 2 | 385.41 | 27.31 |  |  | 2 | 236.2 | 26.09 |  |  | 2 | 234.51 | 25.26 |
|  |  | 3 | 267.78 | 33.79 |  |  | 3 | 228.56 | 26.53 |  |  |  | . | . |
|  |  | 4 | 265.8 | 32.97 |  |  | 4 | 215.2 | 26.48 |  |  |  | . | . |
|  |  | 5 | 277.8 | 32.34 |  |  | 5 | 331.95 | 13.04 |  |  |  | . | . |
|  |  |  | . | . |  |  | 6 | 280.38 | 24.26 |  |  |  | . | . |
|  |  |  | . | . |  |  | 7 | 233.43 | 13.23 |  |  |  | . | . |
|  |  |  | . | . |  |  | 8 | 202.73 | 26.24 |  |  |  | . | . |
|  |  |  | . | . |  |  | 9 | 435.58 | 27.9 |  |  |  | . | . |
| **5** | 3 | 1 | 373.05 | 40.66 | **5** | 2 | 1 | 394.85 | 25.04 | **5** | 2 | 1 | 389.27 | 24.77 |
|  |  | 2 | 216.32 | 35.46 |  |  | 2 | 174.27 | 20.31 |  |  | 2 | 370.52 | 25.07 |
|  |  | 3 | 360.06 | 52.52 |  |  |  | . | . |  |  |  | . | . |
| **6** | 3 | 1 | 278.75 | 29.63 | **6** | 3 | 1 | 357.86 | 18.17 | **6** | 1 | 1 | 562.7 | 28.18 |
|  |  | 2 | 243.67 | 40.43 |  |  | 2 | 285.5 | 25.6 |  |  |  | . | . |
|  |  | 3 | 203.14 | 37.17 |  |  | 3 | 253.9 | 18.33 |  |  |  | . | . |
| **7** | 3 | 1 | 562.47 | 34.26 | **7** | 3 | 1 | 873.19 | 24.49 | **7** | 3 | 1 | 651.71 | 26.75 |
|  |  | 2 | 361.82 | 39.26 |  |  | 2 | 216.3 | 17.97 |  |  | 2 | 397.32 | 27.63 |
|  |  | 3 | 305.43 | 153.9 |  |  | 3 | 226.08 | 19.33 |  |  | 3 | 290.98 | 26.45 |
| **8** | 3 | 1 | 424.91 | 39.86 | **8** | 3 | 1 | 350.83 | 26.03 | **8** | 4 | 1 | 223.46 | 34.22 |
|  |  | 2 | 581.69 | 30.7 |  |  | 2 | 474.77 | 22.44 |  |  | 2 | 262.76 | 28.88 |
|  |  | 3 | 461.01 | 255.2 |  |  | 3 | 380.26 | 17.82 |  |  | 3 | 279.85 | 26.27 |
|  |  |  | . | . |  |  |  | . | . |  |  | 4 | 213.16 | 26.71 |
| **9** | 3 | 1 | 234.75 | 33.06 | **9** | 4 | 1 | 224.96 | 21.46 | **9** | 2 | 1 | 265.23 | 26.96 |
|  |  | 2 | 410.28 | 36.08 |  |  | 2 | 292.98 | 23.32 |  |  | 2 | 425.13 | 27.83 |
|  |  | 3 | 221.93 | 39.41 |  |  | 3 | 193.95 | 24.43 |  |  |  | . | . |
|  |  |  | . | . |  |  | 4 | 188.39 | 24.63 |  |  |  | . | . |
| **10** | 3 | 1 | 325.06 | 48.48 | **10** | 3 | 1 | 206.28 | 22.42 | **10** | 4 | 1 | 247.66 | 27.19 |
|  |  | 2 | 308.32 | 35.95 |  |  | 2 | 181.09 | 18.7 |  |  | 2 | 285.8 | 53.4 |
|  |  | 3 | 273.63 | 36.52 |  |  | 3 | 280.39 | 21.24 |  |  | 3 | 326.81 | 29.56 |
|  |  |  | . | . |  |  |  | . | . |  |  | 4 | 293.7 | 33.82 |
| **11** | 2 | 1 | 313.5 | 32.73 |  |  |  |  |  | **11** | 4 | 1 | 254.7 | 20.73 |
|  |  | 2 | 252.17 | 37.91 |  |  |  |  |  |  |  | 2 | 359.55 | 25.1 |
|  |  |  | . | . |  |  |  |  |  |  |  | 3 | 121.75 | 27.74 |
|  |  |  | . | . |  |  |  |  |  |  |  | 4 | 352.22 | 23.19 |
| **12** | 1 | 1 | 551.25 | 36.4 |  |  |  |  |  | **12** | 3 | 1 | 311.93 | 20.53 |
|  |  |  | . | . |  |  |  |  |  |  |  | 2 | 229.23 | 26.66 |
|  |  |  | . | . |  |  |  |  |  |  |  | 3 | 225.07 | 33.99 |
| **13** | 1 | 1 | 477.01 | 34.65 |  |  |  |  |  |  |  |  |  |  |
| **14** | 2 | 1 | 188.84 | 27.19 |  |  |  |  |  |  |  |  |  |  |
|  |  | 2 | 412.41 | 33.74 |  |  |  |  |  |  |  |  |  |  |
| **Px4-3**  **HSOC 5** |  |  |  |  | **Control HSOC 5** |  |  |  |  | **ETA**  **HSOC 5** |  |  |  |  |
| **1** | 1 | 1 | 182.64 | 21.07 | **1** | 4 | 1 | 284.73 | 31.92 | **1** | 1 | 1 | 696.48 | 30.2 |
|  |  |  | . | . |  |  | 2 | 232.25 | 33.58 |  |  |  | . | . |
|  |  |  | . | . |  |  | 3 | 287.55 | 28.01 |  |  |  | . | . |
|  |  |  | . | . |  |  | 4 | 210.17 | 34.28 |  |  |  | . | . |
| **2** | 3 | 1 | 155.79 | 30.97 | **2** | 4 | 1 | 240.19 | 22.94 | **2** | 2 | 1 | 648.6 | 29.43 |
|  |  | 2 | 130.8 | 37.96 |  |  | 2 | 206.11 | 17.55 |  |  | 2 | 274.35 | 35.77 |
|  |  | 3 | 169.36 | 38.46 |  |  | 3 | 202.55 | 28.33 |  |  |  | . | . |
|  |  |  | . | . |  |  | 4 | 175.38 | 22.01 |  |  |  | . | . |
| **3** | 5 | 1 | 187.69 | 54.99 | **3** | 3 | 1 | 274.95 | 22.94 | **3** | 4 | 1 | 248.36 | 34.88 |
|  |  | 2 | 108.15 | 38.56 |  |  | 2 | 258.47 | 28.41 |  |  | 2 | 214.69 | 34.47 |
|  |  | 3 | 118.72 | 42.84 |  |  | 3 | 229.01 | 26.02 |  |  | 3 | 264.03 | 39.43 |
|  |  | 4 | 174.2 | 36.38 |  |  |  | . | . |  |  | 4 | 357 | 31.17 |
|  |  | 5 | 154.67 | 36.7 |  |  |  | . | . |  |  |  | . | . |
| **4** | 2 | 1 | 206.62 | 34.04 | **4** | 2 | 1 | 255.95 | 22.95 | **4** | 2 | 1 | 379.77 | 29.05 |
|  |  | 2 | 168.91 | 44.9 |  |  | 2 | 220.48 | 27.71 |  |  | 2 | 254.4 | 34.52 |
| **5** | 2 | 1 | 271.91 | 34.36 | **5** | 2 | 1 | 630.57 | 32.77 | **5** | 9 | 1 | 263.15 | 34.55 |
|  |  | 2 | 249.79 | 36.75 |  |  | 2 | 227.72 | 30.87 |  |  | 2 | 237.99 | 37.81 |
|  |  |  | . | . |  |  |  | . | . |  |  | 3 | 370.68 | 36.82 |
|  |  |  | . | . |  |  |  | . | . |  |  | 4 | 229.16 | 33.39 |
|  |  |  | . | . |  |  |  | . | . |  |  | 5 | 367.05 | 31.64 |
|  |  |  | . | . |  |  |  | . | . |  |  | 6 | 203.93 | 36.89 |
|  |  |  | . | . |  |  |  | . | . |  |  | 7 | 530.21 | 33.82 |
|  |  |  | . | . |  |  |  | . | . |  |  | 8 | 245.68 | 35 |
|  |  |  | . | . |  |  |  | . | . |  |  | 9 | 292.01 | 37.85 |
| **6** | 1 | 1 | 440.75 | 54.69 | **6** | 2 | 1 | 221.38 | 26.84 | **6** | 8 | 1 | 230.96 | 32.79 |
|  |  |  | . | . |  |  | 2 | 316.11 | 30.29 |  |  | 2 | 316.67 | 32.68 |
|  |  |  | . | . |  |  |  | . | . |  |  | 3 | 263.97 | 26.62 |
|  |  |  | . | . |  |  |  | . | . |  |  | 4 | 317.52 | 33.13 |
|  |  |  | . | . |  |  |  | . | . |  |  | 5 | 212.13 | 26.98 |
|  |  |  | . | . |  |  |  | . | . |  |  | 6 | 220.26 | 28.2 |
|  |  |  | . | . |  |  |  | . | . |  |  | 7 | 195.54 | 36.99 |
|  |  |  | . | . |  |  |  | . | . |  |  | 8 | 162.73 | 35.49 |
| **7** | 1 | 1 | 241.07 | 35.72 | **7** | 2 | 1 | 228.26 | 26.53 | **7** | 2 | 1 | 457.58 | 35.44 |
|  |  |  | . | . |  |  | 2 | 368.16 | 28.89 |  |  | 2 | 214.21 | 36.5 |
| **8** | 1 | 1 | 306.78 | 42.99 | **8** | 2 | 1 | 714.39 | 29.85 | **8** | 3 | 1 | 291.08 | 29.53 |
|  |  |  | . | . |  |  | 2 | 198.61 | 25.64 |  |  | 2 | 362.73 | 26.89 |
|  |  |  | . | . |  |  |  | . | . |  |  | 3 | 235.19 | 29.14 |
| **9** | 3 | 1 | 150.37 | 35.1 | **9** | 2 | 1 | 423.29 | 20.1 | **9** | 10 | 1 | 366.47 | 29.01 |
|  |  | 2 | 172.15 | 32.28 |  |  | 2 | 305.92 | 16.68 |  |  | 2 | 327.84 | 32.58 |
|  |  | 3 | 265.92 | 45.3 |  |  |  | . | . |  |  | 3 | 189.17 | 27.72 |
|  |  |  | . | . |  |  |  | . | . |  |  | 4 | 192.59 | 27.08 |
|  |  |  | . | . |  |  |  | . | . |  |  | 5 | 353.24 | 32.02 |
|  |  |  | . | . |  |  |  | . | . |  |  | 6 | 269.45 | 28.7 |
|  |  |  | . | . |  |  |  | . | . |  |  | 7 | 382.32 | 29.5 |
|  |  |  | . | . |  |  |  | . | . |  |  | 8 | 246.24 | 29.04 |
|  |  |  | . | . |  |  |  | . | . |  |  | 9 | 253.21 | 30.71 |
|  |  |  | . | . |  |  |  | . | . |  |  | 10 | 280.75 | 30.79 |
| **10** | 2 | 1 | 202.79 | 37.01 | **10** | 5 | 1 | 349.74 | 25.66 | **10** | 1 | 1 | 492.52 | 21.31 |
|  |  | 2 | 245.18 | 33.81 |  |  | 2 | 245.14 | 22.82 |  |  |  | . | . |
|  |  |  | . | . |  |  | 3 | 276.42 | 22.41 |  |  |  | . | . |
|  |  |  | . | . |  |  | 4 | 353.83 | 21 |  |  |  | . | . |
|  |  |  | . | . |  |  | 5 | 233.81 | 20.61 |  |  |  | . | . |
| **11** | 1 | 1 | 318.44 | 40.84 | **11** | 4 | 1 | 195.2 | 24.73 | **11** | 3 | 1 | 296.88 | 39.03 |
|  |  |  | . | . |  |  | 2 | 197.68 | 21.85 |  |  | 2 | 247.76 | 29.11 |
|  |  |  | . | . |  |  | 3 | 216.1 | 22.13 |  |  | 3 | 259.73 | 30.05 |
|  |  |  | . | . |  |  | 4 | 285.36 | 23.85 |  |  |  | . | . |
| **12** | 2 | 1 | 290.59 | 30.24 | **12** | 5 | 1 | 414.2 | 23.19 | **12** | 5 | 1 | 253.63 | 20.69 |
|  |  | 2 | 200.93 | 47.4 |  |  | 2 | 495.76 | 18.21 |  |  | 2 | 221.31 | 34.39 |
|  |  |  | . | . |  |  | 3 | 150.98 | 22.57 |  |  | 3 | 187.11 | 27.46 |
|  |  |  | . | . |  |  | 4 | 374.97 | 22.21 |  |  | 4 | 225.93 | 26.75 |
|  |  |  | . | . |  |  | 5 | 294.91 | 18.97 |  |  | 5 | 171.19 | 28.06 |
| **13** | 4 | 1 | 289.62 | 45.48 | **13** | 3 | 1 | 340.29 | 20.74 | **13** | 9 | 1 | 166.49 | 33.64 |
|  |  | 2 | 194.4 | 51.28 |  |  | 2 | 783.38 | 18.93 |  |  | 2 | 215.44 | 25.28 |
|  |  | 3 | 137.16 | 53.05 |  |  | 3 | 573.22 | 18.72 |  |  | 3 | 216 | 27.63 |
|  |  | 4 | 141.85 | 43.9 |  |  |  | . | . |  |  | 4 | 238.73 | 27.8 |
|  |  |  | . | . |  |  |  | . | . |  |  | 5 | 196.05 | 29.44 |
|  |  |  | . | . |  |  |  | . | . |  |  | 6 | 181.64 | 34.08 |
|  |  |  | . | . |  |  |  | . | . |  |  | 7 | 322.03 | 34.58 |
|  |  |  | . | . |  |  |  | . | . |  |  | 8 | 169.34 | 27.9 |
|  |  |  | . | . |  |  |  | . | . |  |  | 9 | 255.69 | 30.19 |
| **14** | 1 | 1 | 251.77 | 39.78 | **14** | 5 | 1 | 117.09 | 5.098 | **14** | 6 | 1 | 145.19 | 22.34 |
|  |  |  | . | . |  |  | 2 | 345.33 | 21.92 |  |  | 2 | 188.05 | 25.83 |
|  |  |  | . | . |  |  | 3 | 352.33 | 20.01 |  |  | 3 | 261.97 | 25.85 |
|  |  |  | . | . |  |  | 4 | 356.64 | 18.92 |  |  | 4 | 320.44 | 22.83 |
|  |  |  | . | . |  |  | 5 | 258.5 | 18.9 |  |  | 5 | 249.56 | 23.96 |
|  |  |  | . | . |  |  |  | . | . |  |  | 6 | 346.83 | 28.89 |

Desm. Desmosome; Int. Wid. Interdesmosomal widening.
